# RETRACTION: Risk Factors and Clinical Characteristics of Surgical Site Infections in Athletes Undergoing Achilles Tendon Repair Surgery

**DOI:** 10.1111/iwj.70626

**Published:** 2025-04-21

## Abstract

**RETRACTION:** GuoC. , ZhangY. , DongW. , HuangB. , and LiuY. , “Risk Factors and Clinical Characteristics of Surgical Site Infections in Athletes Undergoing Achilles Tendon Repair Surgery,” International Wound Journal 21, no. 3 (2024): e14666, 10.1111/iwj.14666.38420668 PMC10902687

The above article, published online on 29 February 2024, in Wiley Online Library (http://onlinelibrary.wiley.com/), has been retracted by agreement between the journal Editor in Chief, Professor Keith Harding; and John Wiley & Sons Ltd. Following an investigation by the publisher, all parties have concluded that this article was accepted solely on the basis of a compromised peer review process. The editors have therefore decided to retract the article. The authors did not respond to our notice regarding the retraction.

**RETRACTION:** C. Guo , Y. Zhang , W. Dong , B. Huang , and Y. Liu , “Risk Factors and Clinical Characteristics of Surgical Site Infections in Athletes Undergoing Achilles Tendon Repair Surgery,” International Wound Journal 21, no. 3 (2024): e14666, 10.1111/iwj.14666.38420668 PMC10902687

The above article, published online on 29 February 2024, in Wiley Online Library (http://onlinelibrary.wiley.com/), has been retracted by agreement between the journal Editor in Chief, Professor Keith Harding; and John Wiley & Sons Ltd. Following an investigation by the publisher, all parties have concluded that this article was accepted solely on the basis of a compromised peer review process. The editors have therefore decided to retract the article. The authors did not respond to our notice regarding the retraction.

